# Whole-brain connections of glutamatergic neurons in the mouse lateral habenula in both sexes

**DOI:** 10.1186/s13293-024-00611-5

**Published:** 2024-04-23

**Authors:** Hongren Huang, Xue Liu, Liping Wang, Feng Wang

**Affiliations:** 1 Shenzhen Key Laboratory of Neuropsychiatric Modulation, Shenzhen-Hong Kong Institute of Brain Science, Shenzhen Institute of Advanced Technology, Chinese Academy of Sciences, 518055 Shenzhen, China; 2 CAS Key Laboratory of Brain Connectome and Manipulation, the Brain Cognition and Brain Disease Institute, Shenzhen Institute of Advanced Technology, Chinese Academy of Sciences, 518055 Shenzhen, China; 3 Guangdong Provincial Key Laboratory of Brain Connectome and Behavior, the Brain Cognition and Brain Disease Institute, Shenzhen Institute of Advanced Technology, Chinese Academy of Sciences, 518055 Shenzhen, China

**Keywords:** Lateral habenula, Glutamatergic neuron, Sex differences, Connectivity map

## Abstract

**Background:**

The lateral habenula (LHb) is an epithalamus nucleus that is evolutionarily conserved and involved in various physiological functions, such as encoding value signals, integrating emotional information, and regulating related behaviors. The cells in the LHb are predominantly glutamatergic and have heterogeneous functions in response to different stimuli. The circuitry connections of the LHb glutamatergic neurons play a crucial role in integrating a wide range of events. However, the circuitry connections of LHb glutamatergic neurons in both sexes have not been thoroughly investigated.

**Methods:**

In this study, we injected Cre-dependent retrograde trace virus and anterograde synaptophysin-labeling virus into the LHb of adult male and female Vglut2-ires-Cre mice, respectively. We then quantitatively analyzed the input and output of the LHb glutamatergic connections in both the ipsilateral and contralateral whole brain.

**Results:**

Our findings showed that the inputs to LHb^vGlut2^ neurons come from more than 30 brain subregions, including the cortex, striatum, pallidum, thalamus, hypothalamus, midbrain, pons, medulla, and cerebellum with no significant differences between males and females. The outputs of LHb^vGlut2^ neurons targeted eight large brain regions, primarily focusing on the midbrain and pons nuclei, with distinct features in presynaptic bouton across different brain subregions. While correlation and cluster analysis revealed differences in input and collateral projection features, the input-output connection pattern of LHb^vGlut2^ neurons in both sexes was highly similar.

**Conclusions:**

This study provides a systematic and comprehensive analysis of the input and output connections of LHb^vGlut2^ neurons in male and female mice, shedding light on the anatomical architecture of these specific cell types in the mouse LHb. This structural understanding can help guide further investigations into the complex functions of the LHb.

**Supplementary Information:**

The online version contains supplementary material available at 10.1186/s13293-024-00611-5.

## Highlights

The inputs to LHb^vGlut2^ neurons encompass over 30 brain subregions, mainly arising from the pallidum, hypothalamus, and midbrain.

The outputs of LHb^vGlut2^ neurons involve eight large brain regions, mainly targeting midbrain and pons nuclei, displaying distinct features in presynaptic boutons across each subregion.

The pattern of input-output connections of LHb^vGlut2^ neurons is remarkably similar in both males and females.

**Supplementary Information:**

The online version contains supplementary material available at 10.1186/s13293-024-00611-5.

## Plain Language Summary

The glutamatergic neurons in the lateral habenula (LHb) have been implicated in encoding negative emotions and regulating brain functions and psychiatric diseases. However, the extensive brain connections of LHb^vGlut2^ neurons in mice of both sexes have not been comprehensively mapped. Here, we employed cell-type-specific monosynaptic rabies virus tracing to characterize afferent connections onto LHb^vGlut2^ neurons and adeno-associated virus to label glutamatergic efferent axons. Our findings revealed that over 30 brain subregions are involved in the inputs to LHb^vGlut2^ neurons, while the outputs target eight large brain regions. Although correlation and cluster analysis showed variations in inputs and collateral projections, the input-output connection pattern of LHb^vGlut2^ neurons in both sexes was highly similar. Our study provides insights into the anatomical architecture of mouse LHb^vGlut2^ neurons, providing a foundation for further investigations of their complex functions.

## Introduction

The lateral habenula (LHb) is a structure present in vertebrate species that has been extensively studied for its role in connecting the limbic, forebrain, and midbrain regions [1, 2]. It plays a key role in encoding value signals and regulating physiological functions and behaviors [3–5]. The majority of LHb neurons are glutamatergic, expressing *SLC17A6* (*Vglut2*) [3, 6–8], while gamma-aminobutyric acid (GABA)ergic neurons are less abundant [9–12]. These glutamatergic neurons have flexible response capabilities and complex circuitry connections that allow them to alter behaviors and dynamically maintain or modify prior signals [7, 13, 14]. LHb glutamatergic neurons receive inputs from various brain regions, including the ventral pallidum (VP) [15], lateral hypothalamic area (LHA) [16], globus pallidus [17], ventral tegmental area (VTA) [18], and others [5]. These inputs encode value-based, sensory, and experience-dependent information [4]. LHb neurons also send projections to different downstream regions, such as dorsal raphe, to modulate the serotonergic system and depression-related behavioral phenotypes [19, 20].

There is evidence to suggest that LHb circuitry, specific to cell type and/or projection, plays a crucial role in mediating stress-related disorders [21], parental behavior [22], and social communication [23] in a sex-dependent manner. Additionally, cellular and synaptic mechanisms that promote the activity of LHb glutamatergic neurons have been linked to depressive-like behaviors in animal models [24, 25], despite well-documented sex differences in major depressive disorder in clinical reports [26]. It remains to be explored whether there are sex differences in the neural circuits of the LHb^vGlut2^ neurons. Understanding how neural circuits are organized in both sexes is instrumental in comprehending how information is processed in a sex-dependent manner by the LHb. This knowledge could shed light on the potential of LHb as a novel target for therapeutic intervention of affective disorders.

Several studies have utilized anterograde and/or retrograde tracers to investigate the connections of the LHb, either with or without distinguishing between subpopulations of LHb neurons [8, 16, 27–29]. Nonetheless, to date, there is no complete anatomical map of LHb glutamatergic neuron connectivity in mice or any other species. Currently, a comprehensive circuitry connecting the depiction of the LHb^vGlut2^ neurons on a whole-brain scale in both sexes is still lacking.

To address this gap, we utilized cell-type-specific monosynaptic rabies virus tracings to comprehensively identify the whole-brain connectivity map of LHb^vGlut2^ neurons. We also used synaptophysin-expressing adeno-associated viruses to label glutamatergic efferent axons, focusing on presynaptic boutons. Furthermore, we compared the rostro-caudal axis of the connectivity structure of the LHb^vGlut2^ neurons to establish a connectivity-based compartmentalization that may facilitate future functional studies. We also examined and quantified the connectivity of LHb^vGlut2^ neurons in both sexes to increase our understanding of normal brain structure. In summary, we identified input and output subregions of LHb^vGlut2^ neurons and acquired whole-brain quantitative results with specific spatial distribution. We classified the output brain subregions according to the presynaptic boutons and analyzed the input-output connective pattern, which showed distinct clusters but roughly similar connective patterns in both sexes.

## Methods

### Animals

All experimental procedures followed ethical guidelines and have been approved by the Institutional Animal Care and Use Committee at Shenzhen Institute of Advanced Technology (IACUC number: SIAT-IACUC-20,221,209-ZKYSZXJJSYJY-RZC-WF-A2072-02). Adult male and female Vglut2-ires-Cre mice (8–12 weeks old; Jackson lab, Strain#: 016963) used in this study are heterozygous with Cre recombinase under the control of the *vGlut2* gene. The C57BL/6J mice (8–12 weeks old) in the control experiment were purchased from Zhejiang Vital River Laboratory Animal Technology Co., Ltd. (Hangzhou, China). The mice were group-housed at a controlled temperature of 25 degrees Celsius and had free access to food and water. They were also maintained on a 12:12 h light: dark cycle, with lights on from 8:00 a.m. to 8:00 p.m.

### Stereotaxic virus injection

Mice were anesthetized with sodium pentobarbital (100 mg/kg, i.p. injection), and then placed on a stereotaxic apparatus (RWD Co., Ltd., Shenzhen, China). Input tracing experiments were performed on the Vglut2-ires-Cre mice, and C57BL/6J mice were used for the control experiment. Monosynaptic retrograde tracing was achieved by unilaterally injecting a 1:1 mixture of AAV2/9-hSyn-DIO-His-EGFP-2a-TVA-WPRE-pA (viral titer 2.93 × 10^12^ vg/ml; BrainVTA Co., Ltd., Wuhan, China, Cat#PT-0210) and AAV2/9-hSyn-DIO-RVG-WPRE-pA (viral titer 2.98 × 10^12^ vg/ml; BrainVTA Co., Ltd., Wuhan, China, Cat#PT-0204) into the LHb (AP: -1.75 mm; ML: 0.54 mm; DV: -2.75 mm), at a total volume of 15 nl and a flow rate of 30 nl/min. After injection, the microsyringe was kept in place for 10 min before being withdrawn to avoid leakage, and then the skin was sutured. The animals were allowed to recover for 3 weeks after AAV injection. Then, 30 nl of RV-ENVA-△G-dsRed (viral titer 2.00 × 10^8^ infections units/mL; BrainVTA Co., Ltd., Wuhan, China, Cat#R01002) was injected in the same site and expressed for 7 days before the animals were euthanized. To verify the specificity of the viral strategy, we conducted control experiments by injecting a 1:1 mixture of AAV virus encoding TVA and RVG into the LHb in the Vglut2-ires-Cre mice, without RV or followed by injection of RV into the hippocampus 3 weeks later. Alternatively, both TVA/RVG mixture and RV were injected into the LHb in C57BL/6J mice. For mapping inputs of adjacent nucleus, a 1:1 mixture of AAV virus encoding TVA and RVG was injected in the medial habenula (MHb) (AP: -1.75 mm; ML: 0.20 mm; DV: -2.50 mm) or hippocampus (AP: -1.75 mm; ML: 0.55 mm; DV: -2.00 mm) of Vglut2-ires-Cre mice, followed by an injection of RV in the same site 3 weeks later.

For the anterograde axon tracing experiment, AAV2/9-hSyn-FLEX-tdTomato-T2A-Synaptophysin-EGFP-WPRE-pA (viral titer 5.00 × 10^12^ vg/ml; Taitool Co., Ltd., Shanghai, China, Cat#S0161-9) was unilaterally injected into the LHb in Vglut2-ires-Cre mice. The mice were euthanized 4 weeks later, and the fluorescent signals of presynaptic boutons were enhanced through anti-GFP immunostaining.

### Histology and immunostaining

Mice were anesthetized with sodium pentobarbital chloral hydrate (1%, 100 mg/kg, i.p. injection) and then perfused with phosphate-buffered saline (PBS) followed by 4% paraformaldehyde (PFA). The brains were subsequently post-fixed overnight at 4 degrees Celsius in 4% PFA and then cryoprotected in 30% sucrose for 2 days. The brains were sliced into 40 μm coronal sections using a cryostat (Leica, Cat#CM1950), with every third section being used for whole-brain input and output imaging. Cells containing rabies virus were counterstained with DAPI (Sigma-Aldrich, Cat#D9542, 1:5000). For the anterograde tracing experiment, tdTomato and GFP signals located in the presynaptic boutons were immunostained to amplify fluorescent signals of boutons.

For immunostaining, the brain slices were washed with PBS (3 × 10 min) and then permeabilized in a blocking solution (1‰ Triton-X 100, 3% normal donkey serum in PBS) for 1 h. Then, the sections were incubated with primary antibodies, goat anti-GFP (Rockland, Cat#600-101-215, 1:500) and rabbit anti-RFP (Rockland, Cat#600-401-379, 1:500), for 24 h at 4 degrees Celsius. The sections were then washed in PBS (3 × 5 min) and incubated with secondary antibodies, Alexa Fluor-488 donkey anti-goat (Jackson ImmunoResearch, Cat#705-547-003) and Alexa Fluor-594 donkey anti-rabbit (Jackson ImmunoResearch, Cat#711-585-152) for 2 h at room temperature. The sections were then stained with DAPI, washed three times in PBS (10 min per wash), mounted on slides, and sealed with Fluoromount-G (SouthernBiotech, Cat#0100-01) for imaging.

### Imaging acquisition

For whole-brain input and output imaging, the coronal sections were digitally scanned using an automated slide scanner (Olympus, VS120) with a 10 / 0.45 NA objective. For the starter cells imaging, brain slices covering the injection site were acquired through the confocal laser scanning microscope (Olympus, FV3000) with a 40 / 0.95 NA objective. For whole-brain presynaptic boutons imaging and quantification, the same confocal laser scanner was employed to image the innervation of LHb boutons in downstream subregions. Brain slices containing downstream subregions were scanned in a single optical z-section with mosaic stitching covering the structure, and were imaged at a resolution of 0.311 μm/pixel. Three reporter fluorescent proteins (DAPI, EGFP, and tdTomato) were excited by 405 nm, 488 nm, and 594 nm lasers, respectively.

### Counting and data analysis

To delineate the boundaries of each brain subregion, all brain slice images were aligned with the Franklin and Paxinos Atlas (fourth edition) and the Allen Brain Reference Atlas. The quantification of the inputs involved manual counting of starter cells, with only cells displaying distinguishable soma being included in the analysis. The number of input neurons in each brain subregion was summarized by layer, normalized by the total number of input neurons in each sample to determine the input proportion, and then compiled to generate quantified whole-brain inputs.

For measuring the output density, the quantification of synaptophysin-EGFP puncta was semi-automated using ImageJ (NIH, Bethesda, MD, USA). The projection signals were detected by setting the threshold for each section to subtract autofluorescence and segment the binary image in ImageJ. The number of positive pixels within each demarcated area was counted, and the output density percent of brain subregions was calculated by dividing the sum of detected pixels in each section by the total signals across the whole brain, excluding the injection site in each mouse. Bouton characteristics were determined by measuring relative bouton size, and calculating the puncta number and occupied area with ImageJ.

Hierarchical clusters are generated using R and RStudio software (Posit., Boston, MA) to further analyze the output pattern. For classifying presynaptic bouton size, number, and occupied area in each brain subregion, clusters were generated using the “factoextra” package with the “kmeans” method. A hierarchical cluster and heatmap were generated to classify the presynaptic innervating feature of whole-brain subregions based on bouton features including size, number, and occupied area. These features were normalized by the “scale” function. Subsequently, the distance was calculated by the “dist” function, followed by clustering with the “hclust” function, and then a heatmap was generated. These mentioned above classified the innervating characteristic of each brain subregion from the LHb, regardless of sexes.

A Sankey plot was utilized to generate a summary diagram of the input and output connective map using the “networkD3” package. Correlation analysis and clustering of input or output patterns in both sexes were conducted specific steps outlined in a previous reference [30]. A correlation matrix was calculated using the “spearman” method, hierarchical clusters were generated and a heatmap was generated in RStudio to visualize the results.

### Statistical analysis

The data for input proportion and output density were presented as mean ± s.e.m. Differences between groups were analyzed using multiple t-test followed by the Holm-Sidak method for statistical significance. Statistical analyses were conducted using GraphPad Prism 8, and significance levels are indicated as ^*^P < 0.05.

## Results

### Whole brain mapping of the LHb^vGlut2^ input in male and female mice

**Fig. 1:**
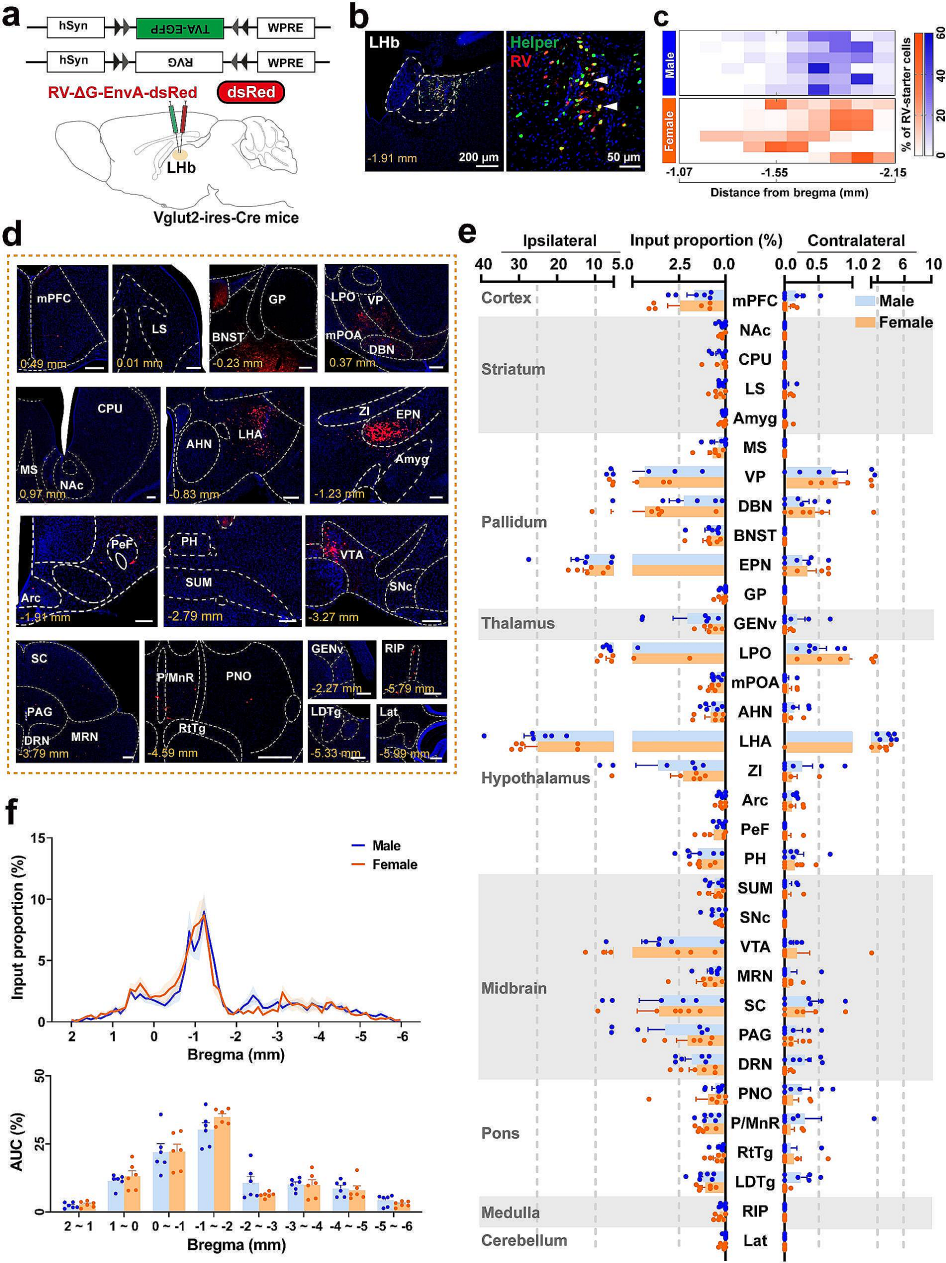
Whole-brain monosynaptic ipsilateral and contralateral inputs to the LHb^vGlut2^ neurons in both sexes. (**a**) Schematic of monosynaptic rabies virus tracing strategy. (**b**) Representative fluorescent micrographs of coronal brain slice containing injection site in the LHb (left) and clear morphology of starter cell soma (right). Scale bar (left) = 200 μm, scale bar (right) = 50 μm. (**c**) Heatmap showing starter cell distribution in each coronal section including LHb of each sample. Each row is from one sample. (**d**) Representative examples of brain-wide ipsilateral (Ipsi) monosynaptic inputs to LHb vGlut2 neurons in one mouse. Scale bars, 200 μm. (**e**) Quantification of ipsilateral and contralateral LHb vGlut2 projecting neurons expressed as a percentage of the total input cells in the whole brains of both male and female mice. (**f**) Input proportion (top) and area under the curve (AUC) (bottom) plots along the anterior-posterior axis covering the entire brain subregions that project to the LHb vGlut2 neurons. Data are shown as mean ± s.e.m., *n* = 6 in each group. The details of abbreviations for brain subregions can be seen in the list of abbreviations

To identify brain-wide inputs to LHb^vGlut2^ neurons, we employed a modified rabies virus-mediated retrograde tracing strategy in the Vglut2-ires-Cre mutant mice (Fig. 1a). The neurons co-labeled by EGFP and dsRed in the injection site were starter cells (Fig. 1b), while dsRed-labeled neurons without EGFP signals were input cells. Most of the starter cells were within the injection site, predominantly located in the central to posterior part of LHb (Fig. 1c; Additional file 1: Fig. S1; Additional file 2: Fig. S2). Cases were only adopted if the starter cells located in the LHb exceeded 70% of the total starter cells (Additional file 3: Fig. S3a). The variability of the retrograde tracing results among different mice was detected by the total number of starter cells. The virus infection efficiency was confirmed by the ratio of total input neurons to the starter cells (Additional file 3: Fig. S3b). Relatively consistent results across different samples indicated a stable virus infection efficiency (Additional file 3: Fig. S3b). Moreover, the specificity of the virus strategy was validated by several control experiments, including the absence of RV (Additional file 3: Fig. S3c, d), the absence of Cre provided by mice (Additional file 3: Fig. S3e), or retrograde labeling targeting in adjacent subregion (Additional file 3: Fig. S3f). These results confirmed that the monosynaptic labeling was specific for vGlut2^+^ neurons in the LHb and that our strategy displayed minimal nonspecific labeling.

We observed retrograde labeling signals in various brain subregions in the ipsilateral and contralateral hemispheres of both sexes, including the cortex, striatum, pallidum, thalamus, hypothalamus, midbrain, pons, medulla, and cerebellum (Fig. 1d, e; Additional file 5: Fig. S5a, c; Additional file 10: Table 1; Additional file 12: Table 3). These findings are consistent with the input circuits to the LHb neurons [27]. We found ipsilateral inputs biased in the inputs of LHb^vGlut2^ neurons, contributing to almost 80% of total inputs, which were distributed mainly in 33 brain subregions throughout the brain (Fig. 1d, e). The inputs from the entopeduncular nucleus (EPN) and LHA contributed the highest proportion of the total input to LHb^vGlut2^ neurons (Fig. 1e). Consistently, the maximum inputs were mainly distributed from bregma − 0.5 to -1.5 mm, where the pallidum and hypothalamus were located (Fig. 1f). We also found that no sex differences were presented in ipsilateral and contralateral inputs across classified groups (Fig. 1e). On the other hand, a small proportion of brain subregions bilaterally innervated the LHb^vGlut2^ neurons, such as the VP, diagonal band nucleus (DBN), lateral preoptic area (LPO), and LHA (Additional file 5: Fig. S5c). We next assessed the ipsilateral and contralateral input connectivity of the LHb^vGlut2^ neurons from 11 large brain regions to gain an overview of the overall connectivity of LHb^vGlut2^ neurons (Additional file 5. Fig. S5a). We found that LHb^vGlut2^ neurons were heavily connected with the pallidum, hypothalamus, and midbrain without showing sex differences.

The habenula region used to be separated into two distinct subregions, the MHb and the LHb. To compare the input pattern with LHb ^vGlut2^ neurons, we explored the inputs of adjacent MHb^vGlut2^ neurons (Additional file 4: Fig. S4a, b). We found that MHb mainly received monosynaptic inputs from the medial septum (MS), DBN, triangular septal nucleus (TS), anterior group of the dorsal thalamus (ATN), interpeduncular nucleus (IPN), and posterodorsal tegmental nucleus (PDTg) (Additional file 4: Fig. S4c, d). Overall, the input map of MHb^vGlut2^ neurons was vastly distinct from the LHb^vGlut2^ neurons.

### Whole brain mapping of the LHb^vGlut2^ output in male and female mice

**Fig. 2:**
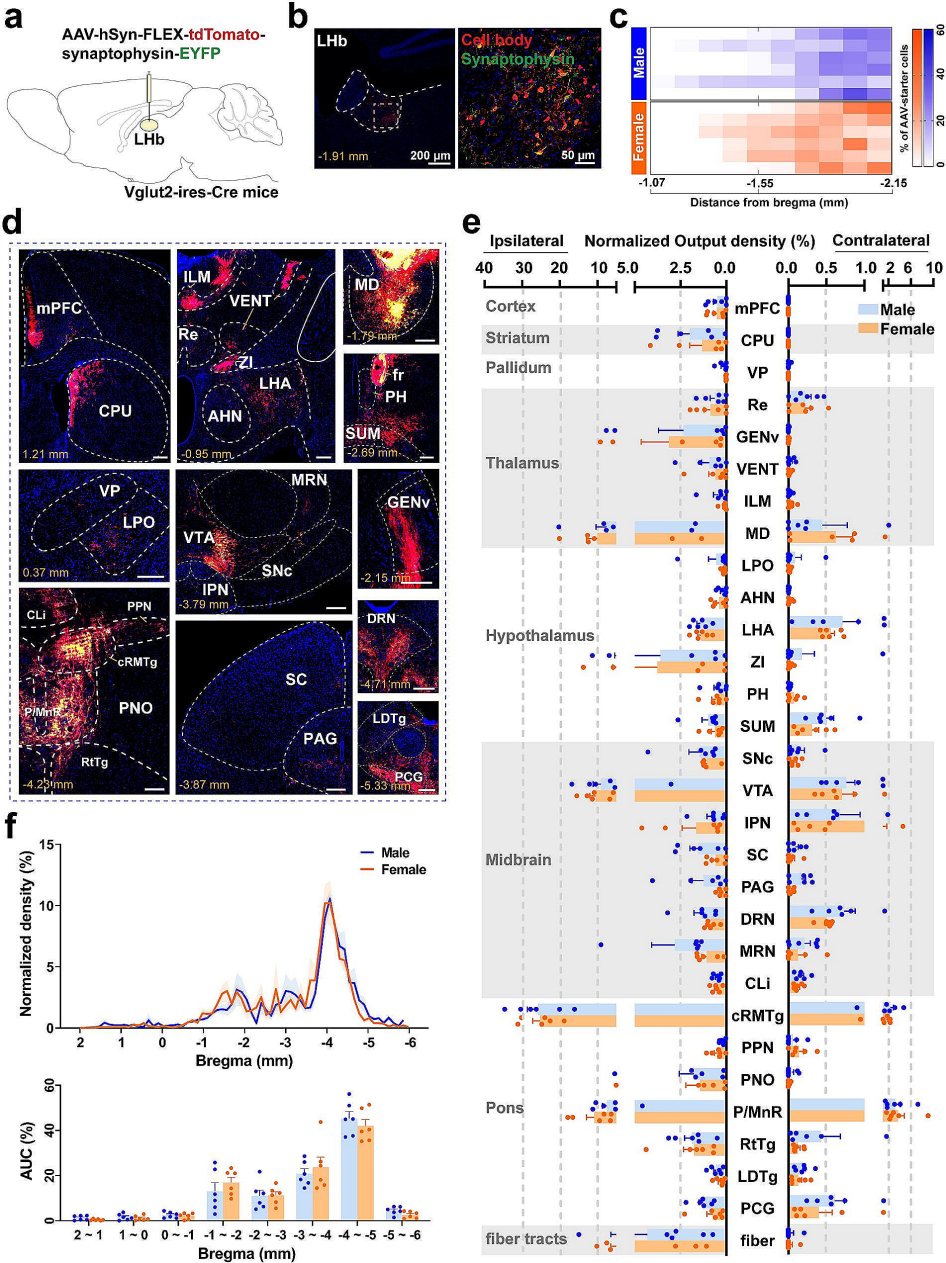
Whole-brain outputs of LHb^vGlut2^ neurons in both sexes. (**a**) Schematic of anterograde tracing strategy labeling outputs of LHb^vGlut2^ neurons. (**b**) Representative image showing the virus injection site in the LHb (left) and clear morphology of starter cell soma (right). Scale bar (left) = 200 μm, scale bar (right) = 50 μm. (**c**) Heatmap showing anterograde-tracing-virus infected cell distribution in each coronal section including LHb of each sample. Each row is from one sample. *n* = 6 in each group. (**d**) Representative examples of brain-wide ipsilateral outputs from LHb vGlut2 neurons in one mouse. Scale bars, 200 μm. (**e**) Quantification of the normalized density of ipsilateral and contralateral downstream brain subregions from LHb vGlut2 neurons in the whole brains of both sexes. (**f**) Normalized density of LHb vGlut2 outputs (top) and area under the curve (AUC) (bottom) from the anterior-posterior axis covering the entire efferent brain regions. The details of abbreviations for brain subregions can be seen in the list of abbreviations

To investigate the whole brain output of LHb^vGlut2^ neurons, we employed an anterograde tracing strategy by injecting the AAV virus expressing presynaptic marker synaptophysin conjugated to EGFP and membrane-bound tdTomato into the unilateral LHb of Vglut2-ires-Cre mice (Fig. 2a). The tdTomato signals were found in the soma (Fig. 2b; Additional file 6: Fig. S6; Additional file 7: Fig. S7) and the downstream axons, while the presynaptic boutons originating from the starter cells were labeled by the EGFP signals (Fig. 2d). The AAV starter cells were predominantly distributed in the central to posterior part of the LHb in both sexes (Fig. 2c).

On a whole brain scale, we found that LHb^vGlut2^ neurons projected to over 30 downstream regions, spanning over seven brain regions and fiber tracts in male and female mice (Fig. 2d; Additional file 11: Table 2). These extensive projections of LHb^vGlut2^ neurons, ranging from the anterior cortex to the caudal pons, targeted voluminous regions of the midbrain and pons area (Additional file 11: Table 2; Additional file 12: Table 3). Moreover, the highest density of outputs consistently arose from bregma − 3.0 to -4.5 mm, where the midbrain and pons are located (Fig. 2f; Additional file 5. Fig. S5b). Regarding the predominant output subregions, the efferent projections were enriched in a specific subset, including the mediodorsal nucleus of the thalamus (MD), VTA, caudal rostral-medial tegmental (cRMTg), and median raphe nucleus (P/MnR) (Fig. 2e). Particularly, the normalized output density of the ipsilateral MD, VTA, cRMTg, and P/MnR, comprising approximately 10%, 10%, 25%, and 10% of the total outputs, respectively (Fig. 2e). Overall, midbrain and pons contributed to the highest projection density, accounting for approximately 70% of the total outputs.

Comparing with the input map of LHb^vGlut2^ neurons, we found there was an absence of efferent to the cerebellum, but a presence of presynaptic boutons from the LHb^vGlut2^ neurons in fiber tracts (Additional file 5. Fig. S5a, b). Similarly, we found no sex differences in output density across all brain subregions, hence the input and output strength of LHb^vGlut2^ neurons between males and females seem comparable.

### Axonal bouton dynamics of LHb^vGlut2^ neurons in the whole brain of both sexes

**Fig. 3:**
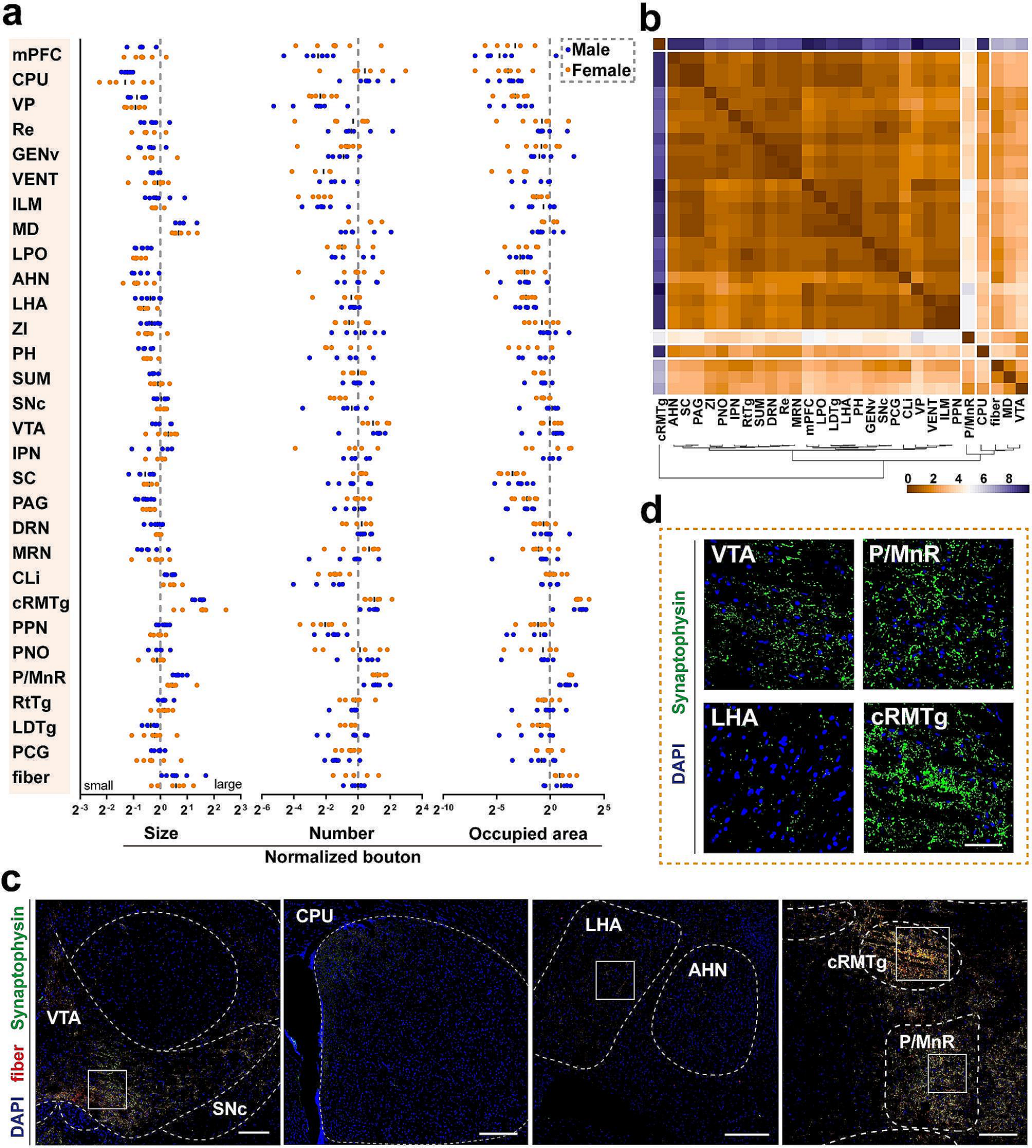
Presynaptic boutons features from LHb^vGlut2^ neurons in the whole brain. (**a**) Quantification of normalized bouton size (left), number (middle), and occupied area (right) in each of the 30 identified target subregions. *n* = 6 in each group. (**b**) Cluster of innervating characteristics of the downstream nucleus including bouton size, number, and occupied area. (**c**) Representative images showing the spatial distribution of presynaptic boutons in subregions from different clusters. Scale bars, 200 μm. (**d**) Representative images showing different presynaptic boutons size from brain subregions of different clusters. Scale bars, 50 μm. The details of abbreviations for brain subregions can be seen in the list of abbreviations

The execution of neuronal function depends on synaptic communication within the cell population [31, 32]. The glutamatergic circuitry of corticocortical and *trans*-thalamic pathways plays a crucial role in information transmission and processing networks in the brain, falling into two main categories [33]. One category conveys information through large synaptic boutons, while the other utilizes small terminal boutons to modulate cortical connections. With distinct features of boutons (Fig. 3a), we classified brain subregions into different clusters according to various features of LHb^vGlut2^ presynaptic boutons, including bouton size (Additional file 8. Fig. S8a), number (Additional file 8. Fig. S8b), and occupied area (Additional file 8. Fig. S8c), respectively, as well as combination of these three features (Fig. 3a, b).

Regarding bouton size, we identified four distinct clusters (Additional file 8. Fig. S8a). Specifically, the cRMTg displayed the largest bouton size among all downstream subregions, while the MD, P/MnR, CLi, and fiber tracts showed relatively large bouton sizes. Conversely, areas such as the mPFC, VP, lateral geniculate nucleus (GENv), and the other nine nuclei showed small bouton size (Fig. 3a, d; Additional file 8. Fig. S8a). The unique morphology of presynaptic boutons indicates that projections from LHb^vGlut2^ neurons may possess diverse functional properties. In addition to bouton size, the efficacy of synaptic functions is also influenced by the number of synapses [34]. Remarkably, we observed a high presence of presynaptic boutons in the caudoputamen (CPU) (Fig. 3a; Additional file 8. Fig. S8a). Moreover, most brain subregions showed small occupied areas, indicating specific spatial characteristics of these efferents from LHb (Fig. 3a; Additional file 8. Fig. S8a).

Combined with these three boutons features collectively, we identified five distinct clusters for these brain-wide subregions (Fig. 3b). Cluster I displayed a large bouton size and relatively moderate distribution, notably in the MD and VTA (Fig. 3c, d). Cluster II showed a relatively small bouton size and restricted spatial location, particularly in the CPU (Fig. 3b, c), indicating potential spatial specificity in the function of this connection. Cluster III displayed a moderate bouton size and broad spatial distribution, such as the P/MnR (Fig. 3b, c, d). Cluster IV demonstrated moderate to dense numbers but tiny boutons, involving regions like the dorsal nucleus raphe (DRN), zona incerta (ZI), LPO, and LHA (Fig. 3b, c, d). Cluster V demonstrated a dense and widespread distribution with a large bouton size, among which the cRMTg is a typically downstream nucleus (Fig. 3b, c, d). These distinct clusters with varying projecting patterns suggested that LHb functions through unique innervating characteristics or different subsets of the neuronal population.

### Whole-brain connectome of LHb^vGlut2^ neurons

**Fig. 4:**
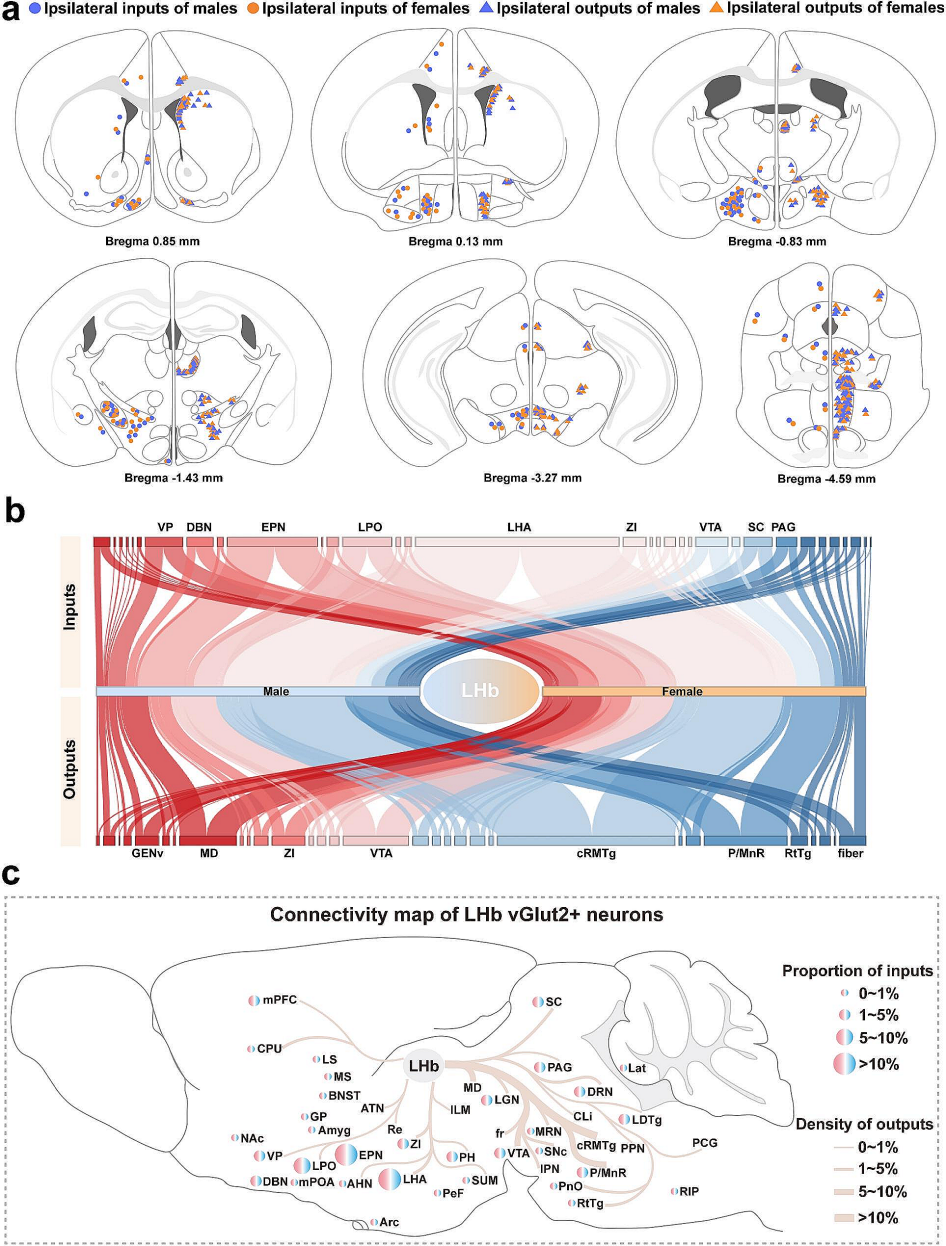
Whole brain LHb^vGlut2^ neurons connectivity map. (**a**) Coronal sections depicting the spatial distribution of ipsilateral inputs and outputs in both sexes. Left, input; right, output; blue dot, male; orange dot, female. (**b**) Sankey network showing the connective strength of subregions (the width of the curve represents connective strength). (**c**) Schematic showing connectivity map of LHb^vGlut2^ neurons. The details of abbreviations for brain regions can be seen in the list of abbreviations

**Fig. 5:**
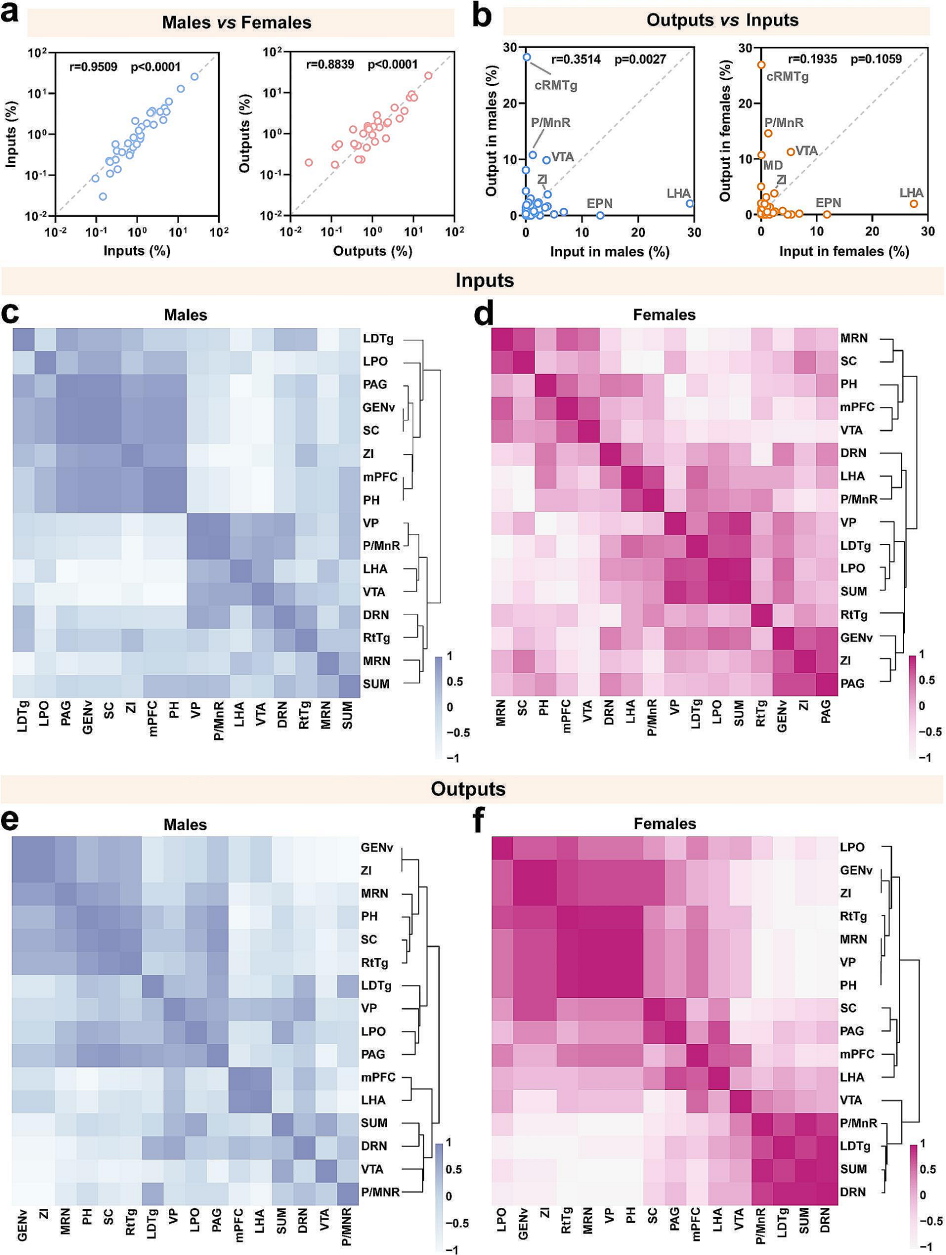
Whole-brain connection pattern of LHb^vGlut2^ connectivity. (**a**) Comparison of inputs (left) and outputs (right) between females and males. (**b**) Comparison of inputs and outputs in male (left) and female (right). (**c**, **d**) Matrices of hierarchically clustered pair-wise correlation coefficients (Spearman) of inputs. Inputs vs. inputs in males (c) or females (d). (**e**, **f**) Matrices of hierarchically clustered pair-wise correlation coefficients (Spearman) of outputs. Outputs vs. outputs in males (e) or females (f). The details of abbreviations for brain subregions can be seen in the list of abbreviations

To establish a refined connective map of LHb^vGlut2^ neurons, we delineated the whole-brain spatial distribution of ipsilateral inputs and outputs of LHb^vGlut2^ neurons in male and female mice (Fig. 4a). Based on the calculated proportion of inputs and outputs in the resided anatomy group, we constructed a whole-brain connection of LHb^vGlut2^ neurons (Fig. 4b, c). Regarding inputs to and outputs from LHb^vGlut2^ neurons, we found that males and females show high similarity with a strong correlation (Fig. 5a). We then investigated the reciprocal connection of the LHb^vGlut2^ neurons by correlating inputs with outputs in male and female mice. However, we found that the majority of brain subregions were often not bidirectionally connected with LHb^vGlut2^ neurons in both sexes (Fig. 5b). Overall, the connective subregions of LHb^vGlut2^ neurons can be classified into three categories: I received reciprocal connection, II only projected to the LHb, and III only received synaptic innervation from the LHb. According to these categories, we then compared input and output patterns in both sexes.

### Comparison of the input and output connection pattern of LHb^vGlut2^ neurons

To explore the similarities and dissimilarities in the connectivity of LHb^vGlut2^ neurons between males and females, we performed a hierarchical cluster analysis of the correlation coefficients. We examined 16 brain subregions with consistent infection efficiency in various samples from both groups for pairwise comparisons (Fig. 5c-f). The cluster data did not show complete consistency in either the inputs or outputs between males and females. The inputs of LHb^vGlut2^ neurons formed obvious but inconsistent clusters in both sexes, suggesting the presence of unique collateral connection patterns (Fig. 5c, d). Furthermore, GENv and mPFC formed separate clusters in both sexes, indicating specific functions associated with these different clusters (Fig. 5c, d). In terms of outputs, GENv and ZI clustered together in both males and females (Fig. 5e, f). Additionally, there were no apparent sex differences in the input and output patterns across the whole brain for male and female mice (Additional file 9. Fig. S9a, b).

## Discussion

In this study, we used monosynaptic retrograde tracing and anterograde tracing to analyze the whole-brain connectivity map of LHb^vGlut2^ neurons in both sexes. We found that the input and output connections of LHb^vGlut2^ neurons are highly similar between males and females. In particular, we identified projections from the LHb^vGlut2^ neurons to the MD, which were neglected previously. Furthermore, we clustered the brain-wide output nuclei into five clusters based on specific presynaptic bouton features, including bouton size, number, and occupied area. Differences in connectivity patterns are related to specific pathways of information processes underlying specific behaviors and functions. Our work lays the groundwork for exploring the relationships between cell heterogeneity of the LHb and anatomical connectivity in specific behavior.

Our study revealed that the LHb^vGlut2^ neurons primarily received inputs from the forebrain [16] and projected to the midbrain [7, 35–37], which is consistent with previous studies. The densest inputs of LHb^vGlut2^ neurons were LHA and EPN, which are closely related to the affective process [38–40]. However, the densest outputs from LHb^vGlut2^ neurons were in the VTA, cRMTg, and P/MnR, which are highly related to the reward process [3, 7, 41, 42]. Furthermore, we found that there were few reciprocal connections of LHb^vGlut2^ neurons, indicating a weak feedback circuit of LHb^vGlut2^ neurons.

Although a previous study reported sexual dimorphism in inputs to the LHb [27], our findings did not reveal significant sex differences in whole-brain inputs to the LHb^vGlut2^ neurons. Interestingly, both males and females exhibited highly similar connective features in terms of upstream and downstream connections in both ipsilateral and contralateral areas. These inconsistencies could be attributed to variations in cell types, injection sites, and injection volumes used in different studies. The differences in injection sites and targeted subpopulations between the two studies led us to speculate that specific subsets of GABAergic neurons or other subpopulations in the LHb might influence the observed sex-specific input patterns. Although this study did not reveal sex differences in connections between the LHb and midbrain, a previous study highlighted sex differences in LHb-induced inhibition of the midbrain dopamine neurons firing in rats, which is reduced in females compared to males [43]. These suggested that sex-specific responses by the LHb electrical stimuli may be influenced by factors such as sex hormones, cell identification, and transcript profiling. Clinically relevant sex differences have been noted in the prevalence or severity of several of the conditions including depression [44], schizophrenia [45], and addiction [46]. Understanding the functionality associated with LHb circuits has broad applications in the field of neurological and mental health.

We also discovered that the connectivity map of LHb^vGlut2^ neurons formed distinct clusters and displayed area-specificity in both sexes. For example, the LHb^vGlut2^ projecting neurons from LHA were mainly distributed in anterior parts but dispersed in nearly all areas of the LHA. However, the LHA presynaptic output boutons from the LHb^vGlut2^ neurons targeted nearly all areas but with relatively sparse density. Similarly, the output boutons to the LPO, ZI, P/MnR, and cRMTg displayed a spatial distribution pattern that covered almost the entire area, which could provide valuable insights into their functions. Moreover, we found that one target of the presynaptic boutons originating from the LHb^vGlut2^ neurons was the anterior lateral part of the MD. Previous studies have indicated that MD is implicated in associative memory encoding and retrieval [47], as well as the transmission of sensory information related to sensations like pain and itch [48], which suggested that the LHb-MD pathway might play a role in regulating memory processing [49, 50].

The characteristics of boutons were thought to be related to electrophysiological functions and synaptic strength in cortical-cortical and cortical-thalamus connection [33, 51, 52]. We observed that few output brain subregions received relatively large presynaptic boutons from the LHb^vGlut2^ neurons, while most output nuclei displayed small presynaptic bouton features. Our findings revealed that the LHb^vGlut2^- VTA, P/MnR, and cRMTg circuits accounted for a relatively large part of total output density with large boutons. Previous studies have demonstrated that the LHb sends direct glutamatergic projections to both GABAergic and dopaminergic neurons in the VTA [9, 53, 54], although they are much sparser than the LHb’s projection to cRMTg. These circuits are involved in carrying information related to aversive stimuli [55], passive and conditioned behavioral avoidance induced by specific paradigm [29, 56, 57]. In addition, we found that the presynaptic boutons of LHb^vGlut2^- DRN, Zl, LPO, and LHA displayed dense numbers but small size and wide volume, indicating that these glutamatergic pathways may play a critical role from LHb^vGlut2^ neurons. Furthermore, the size of synaptic boutons is associated with the diameter of axons [58], which is crucial in determining conduction velocity [59]. It is implied that LHb circuits with larger boutons have the potential to transmit signals more rapidly.

The investigation presented here meticulously compared the connective pattern in both sexes, however, several limitations must be considered. Firstly, the inability to quantify the proportion of each subregion output and reconstruct it due to the lack of volume calculation of the whole-brain output. It is necessary to use more precise imaging methods to create a more comprehensive connection reconstruction of LHb^vGlut2^ neurons. Additionally, glutamatergic cells within the LHb expressed the mRNA of either *vGlut2* or *vGlut3* [60] with heterogeneous functions in response to different stimuli. Our study did not delineate the connectivity map of LHb^vGlut3^ neurons. Furthermore, while we excluded the specific input of the MHb from our statistical results, it is inevitable that some input neurons from both the LHb and MHb were excluded, albeit in small numbers. Moreover, the output strength of weak connection from the LHb may be influenced by common projections from the pretectal regions. It is also important to note that the potential for coincidental association may introduce false positive correlations, and observable correlations may not imply a causal relationship between variables. Therefore, further investigation is essential for experimental validation and interpretation.

### Perspective and insight

This study depicted the whole-brain inputs and outputs, along with the spatial distribution and connective pattern of the LHb^vGlut2^ neurons in both sexes. Furthermore, previously overlooked circuit connections were identified, which could be significant in the physiological functions and pathologies associated with LHb^vGlut2^ neurons. In summary, our anatomical description reinforces the functional role of the LHb as a key hub in complex behaviors and mental disorders. The consistent connections observed in both male and female mice imply that the fundamental organizational principle of the neural network of vGlut2^+^ neurons in the LHb is conserved.

### Electronic supplementary material

Below is the link to the electronic supplementary material.

Supplementary Material 1

Supplementary Material 2

Supplementary Material 3

Supplementary Material 4

Supplementary Material 5

Supplementary Material 6

Supplementary Material 7

Supplementary Material 8

Supplementary Material 9

Supplementary Material 10

Supplementary Material 11

Supplementary Material 12

Supplementary Material 13

## Data Availability

The data that support the findings of this study are available from the corresponding author upon reasonable request.

## Abbreviations

AHN: Anterior hypothalamic nucleus
Amyg: Amygdala
Arc: Arcuate hypothalamic nucleus
ATN: Anterior group of the dorsal thalamus
BNST: Bed nuclei of the stria terminalis
CL: Central lateral nucleus of the thalamus
CLi: Central linear nucleus raphe
CM: Central medial nucleus of the thalamus
CPU: Caudoputamen
cRMTg: caudal rostro-medial tegmental nucleus
DBN: Diagonal band nucleus
DRN: Dorsal nucleus raphe
EPN: Entopeduncular nucleus
fiber: fiber tracts
GENv: Geniculate group, ventral thalamus
GP: Globus pallidus
GRN: Gigantocellular reticular nucleus
ILM: Intralaminar nuclei of the dorsal thalamus
IPN: Interpeduncular nucleus
Lat: Lateral (dentate) cerebellar nucleus
LD: Lateral posterior nucleus of the thalamus
LDTg: Laterodorsal tegmental nucleus
LGN: Lateral geniculate nucleus
LHA: Lateral hypothalamic area
LP: Lateral posterior nucleus of the thalamus
LPO: Lateral preoptic area
LS: Lateral septum
MARN: Magnocellular reticular nucleus
MD: Mediodorsal nucleus of the thalamus
MED: Medial group of the dorsal thalamus
MO: Medial orbital cortex
mPFC: medial prefrontal cortex
MRN: Midbrain reticular nucleus
MS: Medial septum
NAc: Nucleus accumbens
P/MnR: Paramedian and median raphe nucleus
PAG: Periaqueductal gray
PBN: Parabrachial nucleus
PCG: Pontine central gray
PCN: Paracentral nucleus
PDTg: Posterodorsal tegmental nucleus
PeF: Perifornical nucleus
PG: Pontine gray
PH: Posterior hypothalamic nucleus
PNO: Pontine reticular nucleus
PPN: Pedunculopontine nucleus
PRT: Pretectal region
PVT: Paraventricular nucleus of the thalamus
Re: Nucleus of reuniens
RIP: Nucleus raphe magnus
RPO: Nucleus raphe pontis
RtTg: Reticulotegmental nucleus of the pons
SC: Superior colliculus
SNc: Substantia nigra, compact part
SNr: Substantia nigra, reticular part
SUBG: Subgeniculate nucleus
SUM: Supramammillary nucleus
TS: Triangular septal nucleus
VAL: Ventral anterior-lateral complex of the thalamus
VENT: Ventral group of the dorsal thalamus
VM: Ventral medial nucleus of the thalamus
VP: Ventral pallidum
VTA: Ventral tegmental nucleus
ZI: Zona incerta
