# Supplementary material for: Whole-brain connections of glutamatergic neurons in the mouse lateral habenula in both sexes
Source: Biol Sex Differ. 2024 Apr 23;15:37. doi: 10.1186/s13293-024-00611-5 (PMC11036720; doi:10.1186/s13293-024-00611-5)
Supplement: Supplementary file 2 — Supplementary Material 2 [file 13293_2024_611_MOESM2_ESM.docx]

**Additional file 2: Figure S2. The distribution of RV-starter cells of the LHb in females**


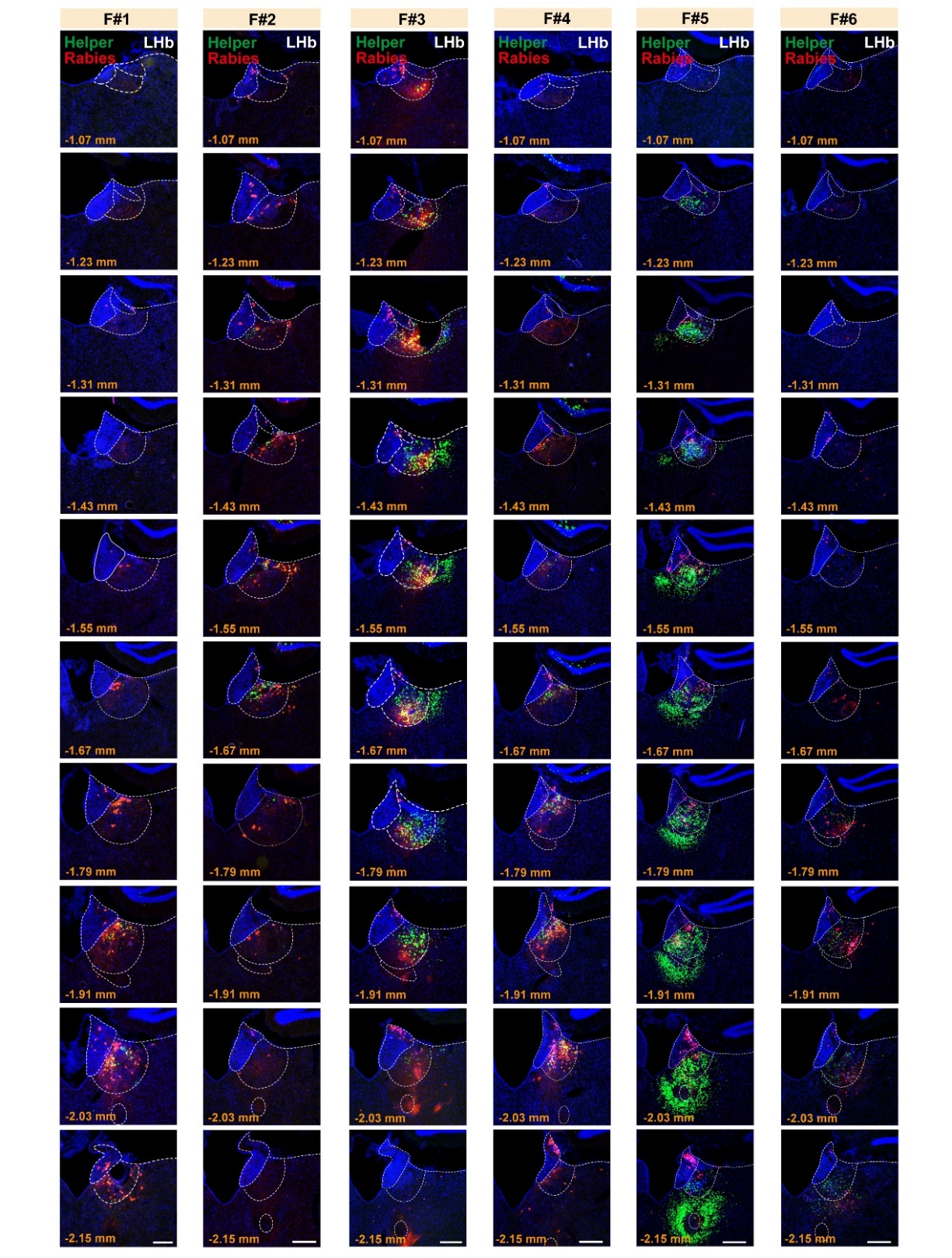


Fluorescent images showing the spatial distribution of RV-starter cells from each sample (row) of females along the anterior (top) – posterior (bottom) axis. dsRed and EGFP co-labeled cells representing RV-starter cells. Scale bar = 200 μm.
