# Supplementary material for: Whole-brain connections of glutamatergic neurons in the mouse lateral habenula in both sexes
Source: Biol Sex Differ. 2024 Apr 23;15:37. doi: 10.1186/s13293-024-00611-5 (PMC11036720; doi:10.1186/s13293-024-00611-5)
Supplement: Supplementary file 3 — Supplementary Material 3 [file 13293_2024_611_MOESM3_ESM.docx]

**Additional file 3: Figure S3. Retrograde tracing to map the inputs to the LHb^vGlut2^ neurons.**


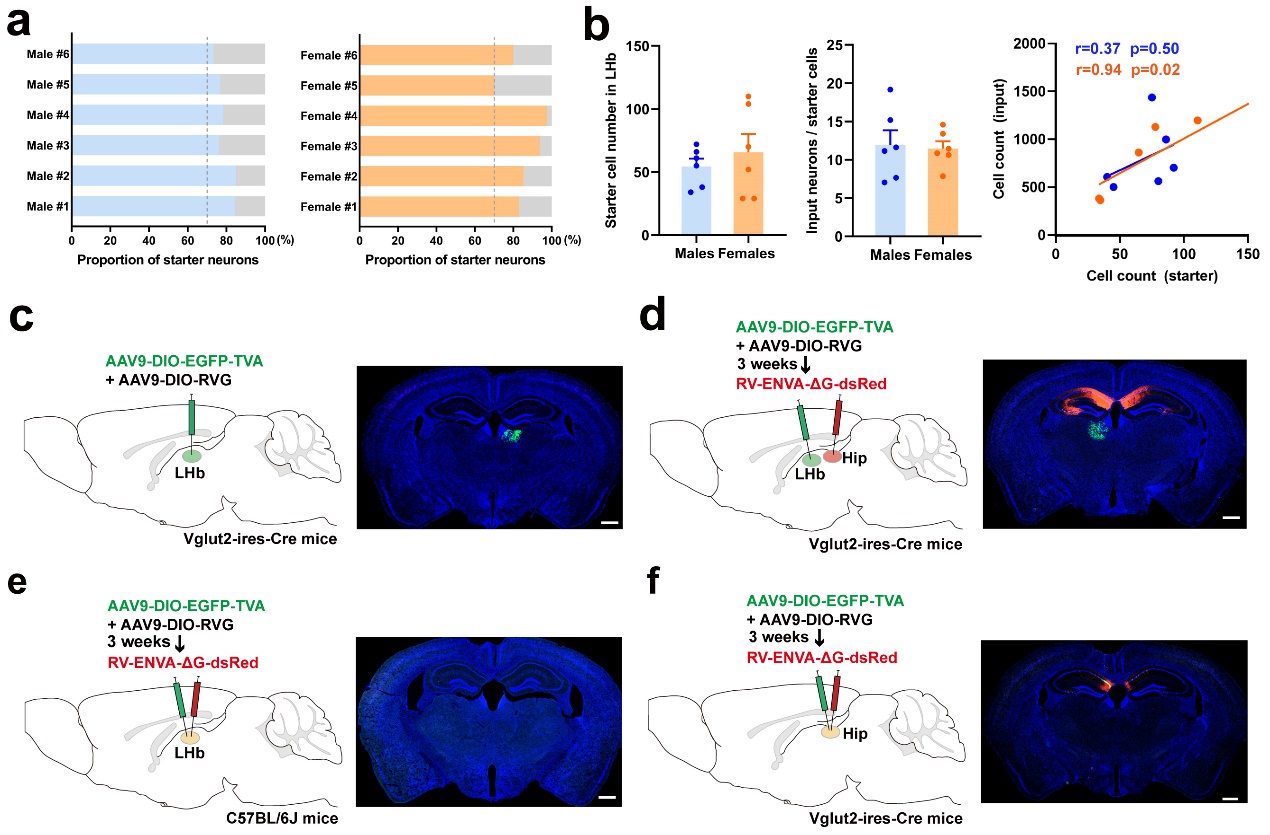


(a) Proportion of starter cells of LHb^vGlut2^ neurons in each sample in males (left) and females (right). (b) Total starter cell number in LHb (left), which was divided into total input number (middle), and linear relationship of starter cell number with total input number (right), indicating the infective efficiency of retrograde-tracing virus in males and females were comparable. Data were shown as mean ± s.e.m. (c-f) Control experiments for the specificity of retrograde tracing strategy. Absence of RV-labeled neurons in AAV-only negative control without rabies virus infection (c). AAVs labeled LHb^vGlut2^ neurons with RV in the hippocampus displayed the absence of LHb afferent inputs (d). AAVs and rabies injected in C57BL/6J mice displayed the absence of LHb afferent inputs (e). The absence of LHb afferent inputs with starter cells is limited within the hippocampus (f). n=3 in each group. Scale bar = 500 μm.
