# Supplementary material for: Whole-brain connections of glutamatergic neurons in the mouse lateral habenula in both sexes
Source: Biol Sex Differ. 2024 Apr 23;15:37. doi: 10.1186/s13293-024-00611-5 (PMC11036720; doi:10.1186/s13293-024-00611-5)
Supplement: Supplementary file 4 — Supplementary Material 4 [file 13293_2024_611_MOESM4_ESM.docx]

**Additional file 4: Figure S4. The input map of the MHb^vGlut2^ neurons.**


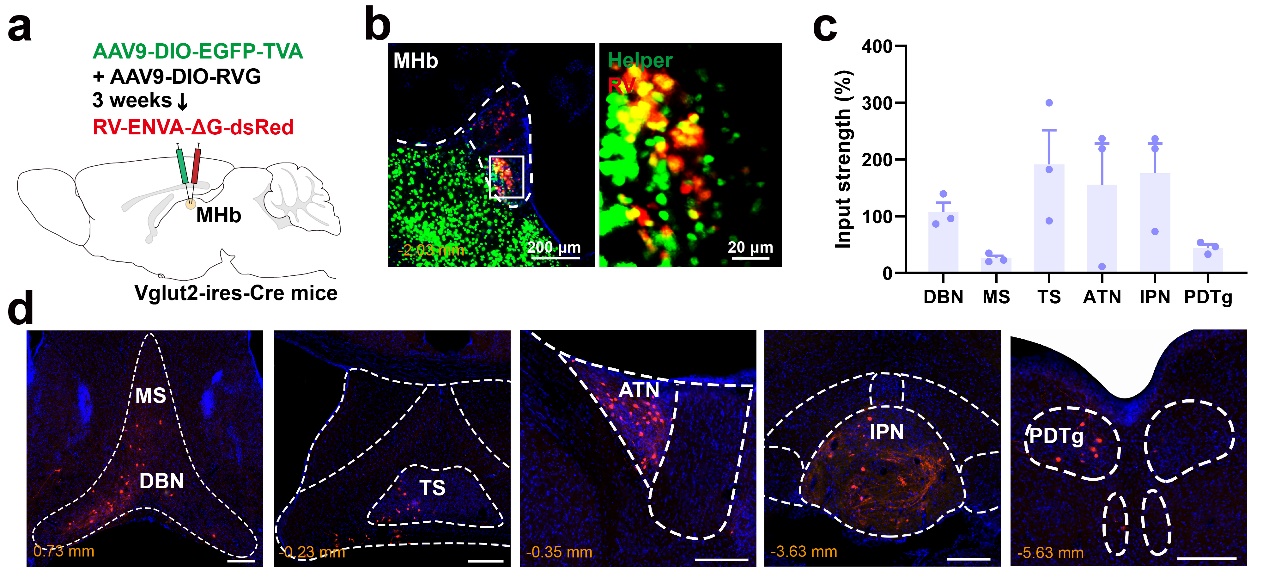


(a) Virus strategy for retrograde labeling vGlut2 positive neurons in the MHb. (b) Representative image of the injection site. Starter cells were highly limited to the MHb. Scale bar (left) = 200 μm; scale bar (right) = 20 μm. (c) The percentage of input strength of the MHb. n = 3 in total. (d) Representative image showing input brain regions to the MHb vGlut2 neurons. scale bar = 200 μm.
