# Supplementary material for: Whole-brain connections of glutamatergic neurons in the mouse lateral habenula in both sexes
Source: Biol Sex Differ. 2024 Apr 23;15:37. doi: 10.1186/s13293-024-00611-5 (PMC11036720; doi:10.1186/s13293-024-00611-5)
Supplement: Supplementary file 5 — Supplementary Material 5 [file 13293_2024_611_MOESM5_ESM.docx]

**Additional file 5: Figure S5. Whole-brain connections of the LHb^vGlut2^ neurons.**


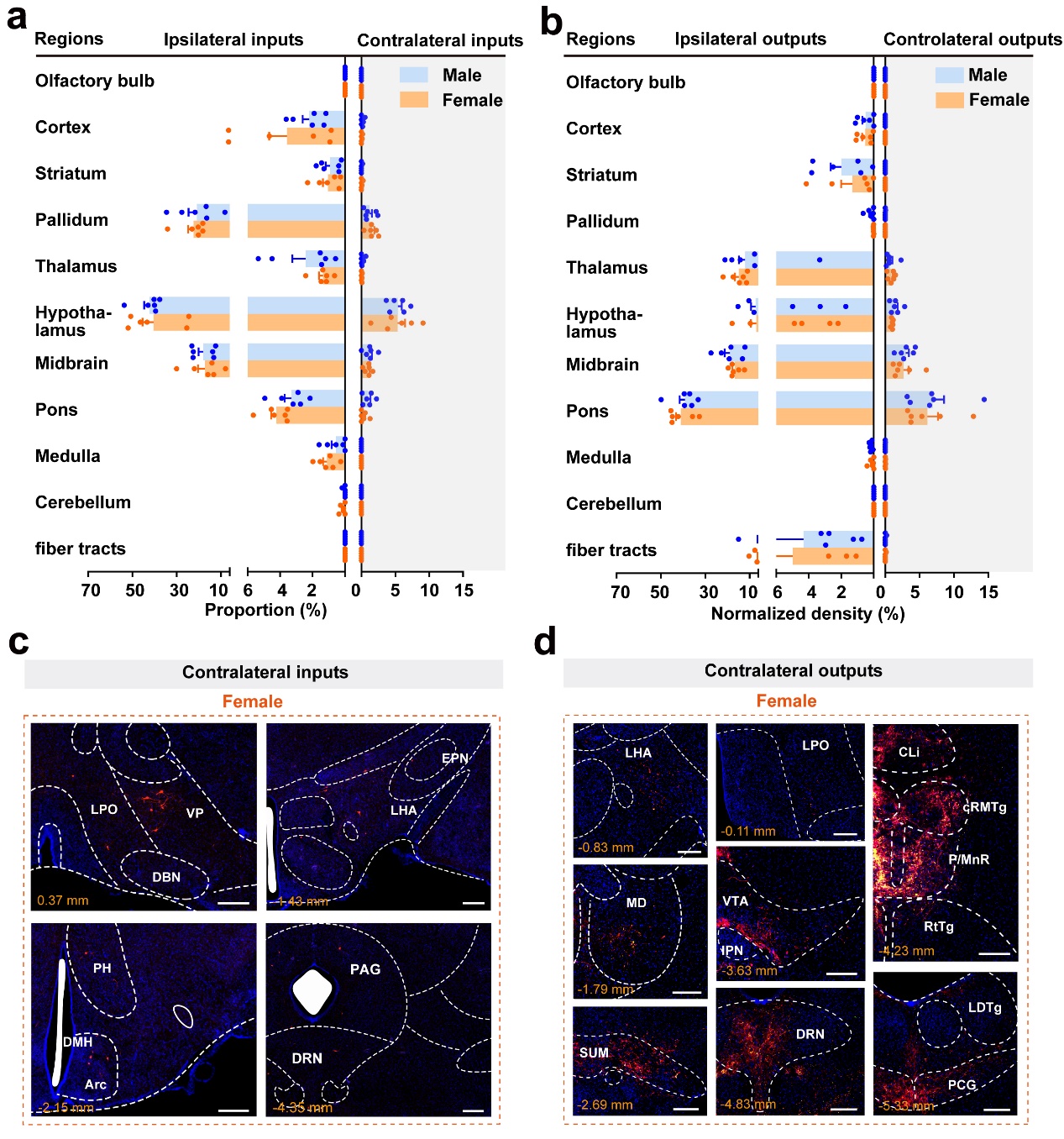


(a, b) The proportion or density of inputs (a) and outputs (b) in the ipsilateral and contralateral side of 11 brain regions. Data are shown as mean ± s.e.m., n = 6. (c, d) Representative image showing contralateral inputs (c) or outputs (d) brain regions of the LHb^vGlut2^ neurons. Scale bars = 200 μm.
