# Supplementary material for: Whole-brain connections of glutamatergic neurons in the mouse lateral habenula in both sexes
Source: Biol Sex Differ. 2024 Apr 23;15:37. doi: 10.1186/s13293-024-00611-5 (PMC11036720; doi:10.1186/s13293-024-00611-5)
Supplement: Supplementary file 6 — Supplementary Material 6 [file 13293_2024_611_MOESM6_ESM.docx]

**Additional file 6: Figure S6. Spatial distribution of AAV starter cells in the LHb in males.**


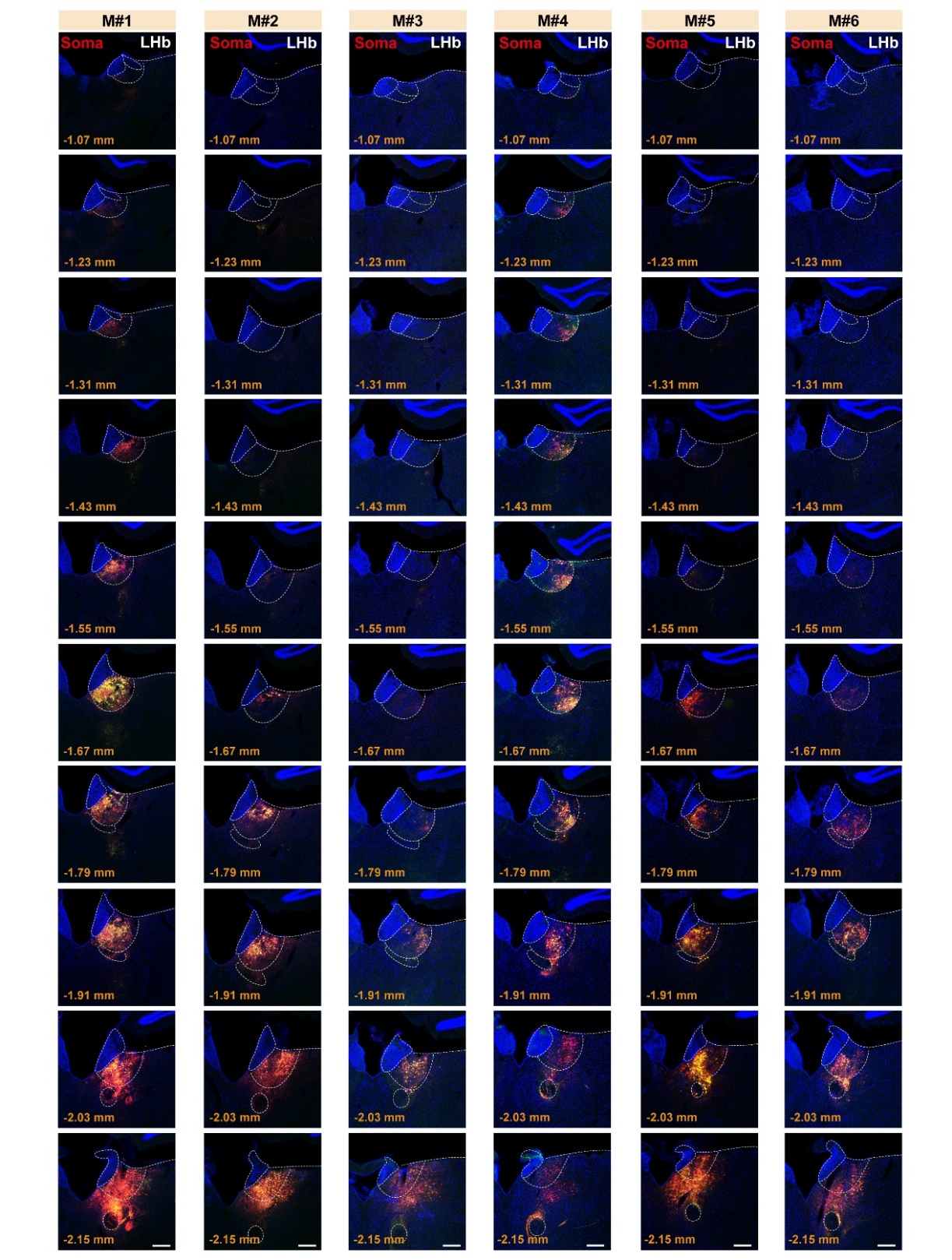


Fluorescent images from each sample of male group showing the spatial distribution of AAV-starter cells. Each row represents the injection site from a sample along the anterior (top) – posterior (bottom) axis. Scale bar = 200 μm.
