# Supplementary material for: Whole-brain connections of glutamatergic neurons in the mouse lateral habenula in both sexes
Source: Biol Sex Differ. 2024 Apr 23;15:37. doi: 10.1186/s13293-024-00611-5 (PMC11036720; doi:10.1186/s13293-024-00611-5)
Supplement: Supplementary file 8 — Supplementary Material 8 [file 13293_2024_611_MOESM8_ESM.docx]

**Additional file 8: Figure S8. Output cluster of LHb^vGlut2^ neurons according to the presynaptic bouton size, number, and occupied area.**
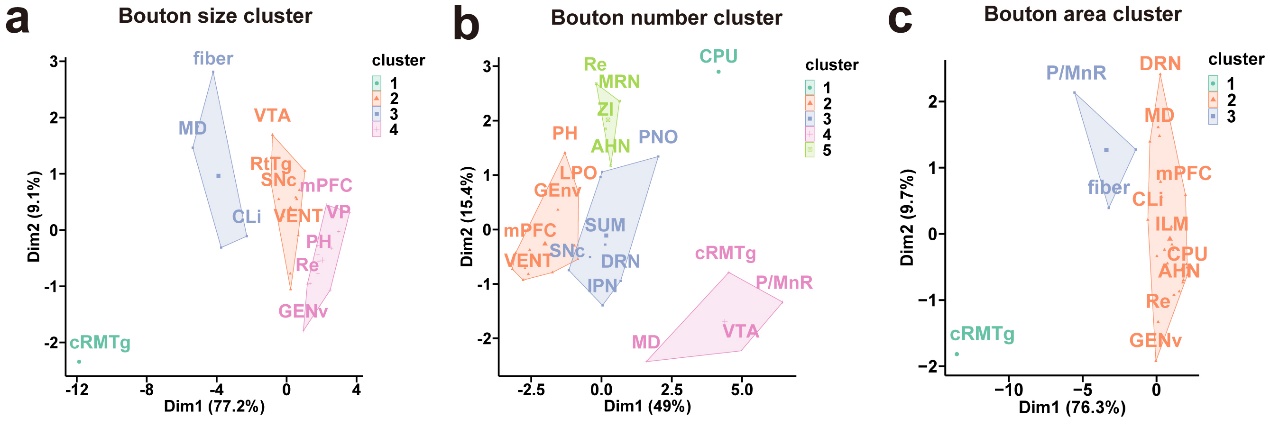


(a-c) Output brain subregions cluster of the LHb^vGlut2^ neurons according to the presynaptic bouton size (a), number (b), and occupied area (c).
