# Supplementary material for: Whole-brain connections of glutamatergic neurons in the mouse lateral habenula in both sexes
Source: Biol Sex Differ. 2024 Apr 23;15:37. doi: 10.1186/s13293-024-00611-5 (PMC11036720; doi:10.1186/s13293-024-00611-5)
Supplement: Supplementary file 9 — Supplementary Material 9 [file 13293_2024_611_MOESM9_ESM.docx]

**Additional file 9: Figure S9.** **Connection pattern of output and input of LHb^vGlut2^ neurons in male and female mice.**


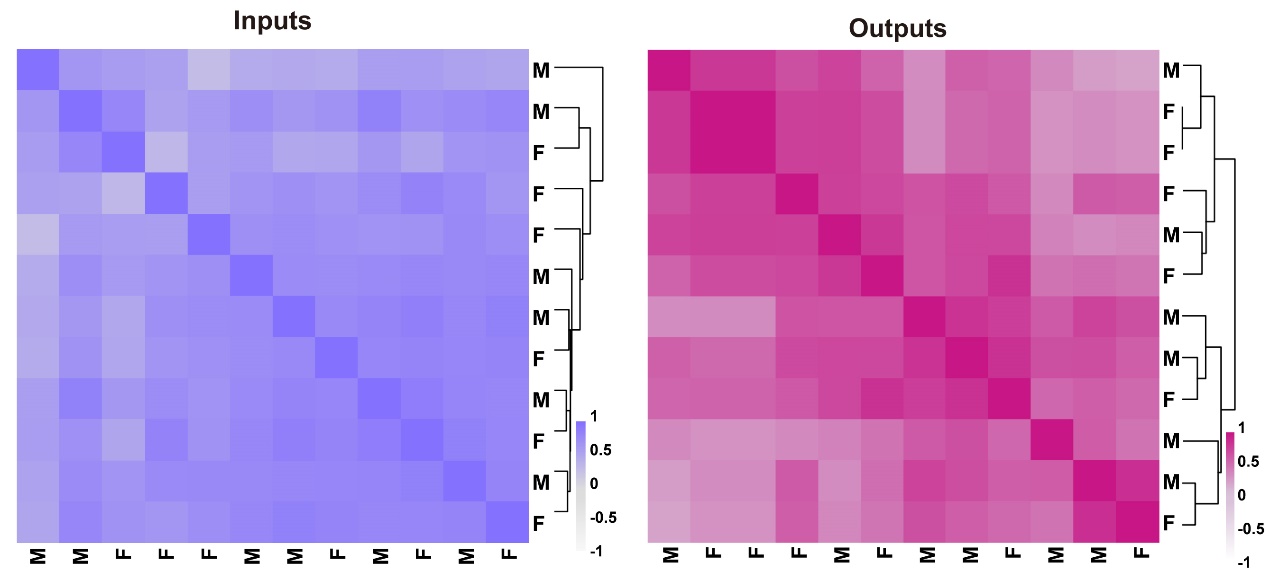


(a-b) Input (a) and output (b) pattern of male and female mice.
