# Supplementary material for: Whole-brain connections of glutamatergic neurons in the mouse lateral habenula in both sexes
Source: Biol Sex Differ. 2024 Apr 23;15:37. doi: 10.1186/s13293-024-00611-5 (PMC11036720; doi:10.1186/s13293-024-00611-5)
Supplement: Supplementary file 11 — Supplementary Material 11 [file 13293_2024_611_MOESM11_ESM.docx]

**Additional file 10: Table 1. The proportion of whole-brain input of the vGlut2 neurons in the LHb**

| Brain area | Subregion | Input proportion in males | Input proportion in females |
| --- | --- | --- | --- |
| Olfactory areas |  | NA | NA |
| Cereberal cortex | Anterior cingulate area | 1.85 ± 1.01 | 2.51 ± 1.47 |
|  | Motor cortex | 0.03 ± 0.07 | 0.23 ± 0.48 |
|  | Visual cortex | 0.14 ± 0.10 | 0.07 ± 0.11 |
|  | Insular cortex | 0.13 ± 0.15 | 0.38 ± 0.70 |
|  | Somatosensory cortex | 0.05 ± 0.07 | 0.06 ± 0.14 |
|  | Orbital cortex | 0.08 ± 0.14 | 0.30 ± 0.27 |
|  | Retrosplenial area | 0.05 ± 0.07 | 0.04 ± 0.10 |
|  | Ectorhinal area | 0.04 ± 0.08 | 0.04 ± 0.05 |
| Striatum | Nucleus accumbens | 0.22 ± 0.19 | 0.20 ± 0.12 |
|  | Caudoputamen | 0.39 ± 0.33 | 0.31 ± 0.47 |
|  | Lateral septal nucleus | 0.27 ± 0.18 | 0.43 ± 0.35 |
|  | Amygdalar nucleus | 0.08 ± 0.08 | 0.17 ± 0.14 |
| Pallidum | Medial septal nucleus | 0.38 ± 0.49 | 0.70 ± 0.52 |
|  | Substantia innominata | 5.03 ± 2.07 | 5.45 ± 1.38 |
|  | Diagonal band nucleus | 2.50 ± 1.98 | 4.78 ± 3.28 |
|  | Bed nuclei of  the stria terminalis | 0.83 ± 0.62 | 0.94 ± 0.61 |
|  | Entopeduncular nucleus | 13.25 ± 7.46 | 11.86 ± 3.79 |
|  | Globus pallidus | 0.11 ± 0.15 | 0.22 ± 0.23 |
| Thalamus | Nucleus of reuniens | 0.11 ± 0.13 | 0.09 ± 0.13 |
|  | Geniculate group,  ventral thalamus | 2.25 ± 1.95 | 0.92 ± 0.50 |
|  | Paraventricular nucleus  of the thalamus | 0.25 ± 0.23 | 0.32 ± 0.27 |
|  | Ventral medial nucleus  of the thalamus | NA | 0.02 ± 0.03 |
|  | Mediodorsal nucleus  of thalamus | 0.02 ± 0.03 | 0.08 ± 0.13 |
| Hypotha-lamus | Lateral preoptic area | 6.77 ± 1.02 | 6.89 ± 2.8 |
|  | Medial preoptic area | 0.50 ± 0.27 | 0.85 ± 0.33 |
|  | Zona incerta | 3.89 ± 2.98 | 2.39 ± 1.69 |
|  | Suprachiasmatic nucleus | NA | 0.13 ± 0.18 |
|  | Anterior hypothalamic nucleus | 0.93 ± 0.40 | 0.88 ± 0.76 |
|  | Paraventricular  hypothalamic nucleus | 0.08 ± 0.08 | 0.16 ± 0.12 |
|  | Ventromedial  hypothalamic nucleus | 0.65 ± 0.41 | 1.54 ± 2.27 |
|  | Dorsomedial nucleus  of the hypothalamus | 0.96 ± 0.31 | 0.95 ± 0.57 |
|  | Lateral hypothalamic area | 29.23 ± 6.16 | 27.49 ± 8.15 |
|  | Arcuate hypothalamic nucleus | 0.32 ± 0.18 | 0.33 ± 0.18 |
|  | Perifornical nucleus | 0.30 ± 0.23 | 0.68 ± 0.59 |
|  | Supramammillary nucleus | 0.66 ± 0.29 | 0.66 ± 0.53 |
|  | Posterior hypothalamic nucleus | 1.67 ± 0.95 | 1.42 ± 0.53 |
| Midbrain | Substantia nigra, reticular part | 0.47 ± 0.55 | 0.33 ± 0.42 |
|  | Substantia nigra, compact part | 0.56 ± 0.44 | 0.34 ± 0.12 |
|  | Ventral tegmental area | 3.69 ± 2.03 | 5.36 ± 4.13 |
|  | Pretectal region | 2.43 ± 1.35 | 1.91 ± 2.36 |
|  | Midbrain reticular nucleus | 0.86 ± 0.69 | 1.24 ± 0.92 |
|  | Superior colliculus | 3.85 ± 2.78 | 3.88 ± 2.93 |
|  | Periaqueductal gray | 3.43 ± 2.18 | 2.19 ± 1.26 |
|  | Dorsal nucleus raphe | 2.13 ± 0.79 | 1.59 ± 0.88 |
|  | Paratrochlear nucleus | 0.07 ± 0.15 | NA |
|  | Central linear nucleus raphe | 0.11 ± 0.11 | 0.06 ± 0.10 |
|  | Inferior colliculus | 0.45 ± 0.40 | 0.32 ± 0.27 |
|  | Edinger-Westphal nucleus | 0.27 ± 0.16 | 0.39 ± 0.31 |
|  | Cuneiform nucleus | 0.75 ± 0.48 | 0.24 ± 0.23 |
| Pons | Caudal rostro-medial  tegmental nucleus | 0.18 ± 0.21 | 0.05 ± 0.07 |
|  | Pedunculopontine nucleus | NA | 0.02 ± 0.03 |
|  | Pontine reticular nucleus | 0.83 ± 0.51 | 1.08 ± 1.57 |
|  | Paramedian and median  raphe nucleus | 1.24 ± 0.92 | 1.31 ± 0.35 |
|  | Reticulotegmental nucleus  of the pons | 0.45 ± 0.33 | 0.65 ± 0.37 |
|  | Laterodorsal tegmental nucleus | 1.45 ± 0.64 | 1.11 ± 0.4 |
|  | Pontine central gray | 0.13 ± 0.14 | 0.05 ± 0.1 |
|  | Pontine reticular nucleus,  caudal part | NA | 0.14 ± 0.14 |
|  | Parabrachial nucleus | 0.05 ± 0.07 | 0.21 ± 0.30 |
| Medulla | Nucleus raphe magnus | 0.14 ± 0.15 | 0.33 ± 0.23 |
|  | Parvicellular reticular nucleus | NA | 0.03 ± 0.07 |
|  | Magnocellular reticular nucleus | 0.17 ± 0.21 | 0.15 ± 0.10 |
|  | Gigantocellular reticular nucleus | 0.12 ± 0.20 | 0.18 ± 0.23 |
|  | Intermediate reticular nucleus | NA | 0.02 ± 0.03 |
|  | Medial vestibular nucleus | 0.03 ± 0.07 | 0.21 ± 0.32 |
|  | Paragigantocellular  reticular nucleus | 0.05 ± 0.07 | 0.03 ± 0.04 |
| Cerebellum |  | 0.03 ± 0.07 | 0.14 ± 0.14 |

Data are shown as mean ± s.e.m. NA represented non observable input neurons. n = 6 per group.
