# Supplementary material for: Whole-brain connections of glutamatergic neurons in the mouse lateral habenula in both sexes
Source: Biol Sex Differ. 2024 Apr 23;15:37. doi: 10.1186/s13293-024-00611-5 (PMC11036720; doi:10.1186/s13293-024-00611-5)
Supplement: Supplementary file 12 — Supplementary Material 12 [file 13293_2024_611_MOESM12_ESM.docx]

**Additional file 11: Table 2. Output density across the whole brain.**

| Mice  Area | | F#1 | F#2 | F#3 | F#4 | F#5 | F#6 | M#1 | M#2 | M#3 | M#4 | M#5 | M#6 |
| --- | --- | --- | --- | --- | --- | --- | --- | --- | --- | --- | --- | --- | --- |
| Olfactory areas |  | NA | NA | NA | NA | NA | NA | NA | NA | NA | NA | NA | NA |
| Cerebral cortex | MO | + | NA | + | + | NA | + | + | NA | NA | + | NA | NA |
|  | mPFC | ++ | + | ++ | + | + | + | + | + | + | ++ | + | ++ |
| Striatum | CPU | ++ | + | + | + | + | ++ | + | + | ++ | ++ | ++ | ++ |
|  | NAc | + | + | NA | + | NA | + | + | NA | + | + | + | + |
| Pallidum | VP | + | + | + | + | + | + | + | + | + | + | + | + |
|  | DBN | + | NA | + | + | NA | + | NA | NA | NA | + | NA | + |
|  | GP | + | + | + | + | NA | + | + | + | + | + | + | + |
| Thalamus  -VENT | VM | + | NA | + | NA | + | + | + | NA | + | + | NA | + |
|  | VAL | + | + | + | + | + | + | + | + | + | + | + | + |
| Thalamus  -LAT | LP | + | + | NA | - | - | + | NA | + | + | NA | + | + |
| Thalamus  -ATN | LD | + | ++ | + | + | + | + | + | ++ | ++ | + | + | + |
| Thalamus  -MED | MD | +++ | ++ | +++ | ++ | +++ | +++ | +++ | ++ | +++ | +++ | ++ | +++ |
| Thalamus  -ILM | PCN | + | + | + | + | + | + | + | + | + | + | + | + |
|  | CL | + | + | + | + | + | + | + | + | + | + | + | + |
|  | CM | + | + | + | + | + | + | + | NA | NA | + | + | + |
| Thalamus  -MTN | PVT | + | + | + | + | + | + | + | + | + | + | + | + |
|  | Re | + | ++ | + | ++ | + | ++ | + | ++ | ++ | + | + | + |
| Thalamus  -GENv | LGN | + | ++ | + | ++ | + | + | + | ++ | ++ | + | + | + |
|  | SUBG | + | ++ | + | ++ | + | ++ | + | +++ | ++ | + | + | + |
| Hypothalamus | LPO | + | + | + | + | + | + | + | + | + | + | ++ | + |
|  | AHN | + | + | + | + | - | + | + | + | + | + | + | + |
|  | LHA | ++ | ++ | + | ++ | ++ | ++ | + | ++ | ++ | ++ | ++ | ++ |
|  | ZI | ++ | +++ | + | +++ | + | +++ | + | +++ | +++ | + | + | ++ |
|  | PH | + | + | + | ++ | + | + | + | ++ | + | + | + | + |
|  | SUM | + | + | + | + | + | + | + | + | + | + | ++ | + |
| Midbrain | SNr | + | NA | NA | NA | NA | + | NA | NA | + | + | NA | + |
|  | VTA | +++ | +++ | +++ | +++ | +++ | +++ | +++ | ++ | +++ | +++ | +++ | +++ |
|  | MRN | ++ | ++ | + | ++ | + | ++ | ++ | ++ | ++ | ++ | ++ | ++ |
|  | SC | + | ++ | + | ++ | + | + | + | ++ | ++ | + | ++ | ++ |
|  | PAG | + | + | + | + | + | + | + | ++ | + | + | ++ | + |
|  | PRT | + | ++ | + | ++ | + | ++ | + | ++ | + | + | + | + |
|  | EW | + | + | + | + | + | + | + | + | + | + | + | + |
|  | SNc | ++ | ++ | ++ | ++ | + | + | + | + | + | ++ | ++ | ++ |
|  | cRMTg | +++ | +++ | +++ | +++ | +++ | +++ | +++ | +++ | +++ | +++ | +++ | +++ |
|  | PPN | + | ++ | + | + | + | + | + | + | + | + | + | + |
|  | IPN | + | + | + | ++ | ++ | + | ++ | + | + | + | + | + |
|  | CLi | + | + | + | + | + | + | + | + | + | + | + | + |
|  | DRN | ++ | ++ | + | + | ++ | + | ++ | + | + | ++ | ++ | ++ |
| Pons | P/MnR | +++ | +++ | +++ | +++ | +++ | +++ | +++ | +++ | +++ | ++ | +++ | +++ |
|  | PNO | + | ++ | + | ++ | + | ++ | + | +++ | ++ | + | ++ | ++ |
|  | PG | + | + | + | + | + | + | + | + | + | + | + | + |
|  | PBN | + | + | + | + | + | + | + | NA | + | + | + | + |
|  | RtTg | ++ | ++ | + | ++ | + | ++ | + | ++ | ++ | ++ | ++ | ++ |
|  | PCG | + | ++ | + | + | + | + | + | + | + | ++ | ++ | ++ |
|  | LDTg | + | + | + | + | + | + | + | + | + | + | ++ | + |
|  | RPO | + | + | + | + | + | + | + | + | + | + | + | + |
| Medulla | GRN | + | + | + | + | + | + | NA | + | + | + | + | + |
|  | MARN | + | + | + | + | NA | + | + | + | + | + | + | + |
| Cerebellum |  | NA | NA | NA | NA | NA | NA | NA | NA | NA | NA | NA | NA |
| fiber tracts | fiber | +++ | +++ | +++ | ++ | ++ | ++ | +++ | + | ++ | ++ | ++ | ++ |

The proportion of normalized output density: +++ >5%, ++ mean > 1~5%, + 0~ 1%, NA means non observable. n = 6 per group.
