# Supplementary material for: Whole-brain connections of glutamatergic neurons in the mouse lateral habenula in both sexes
Source: Biol Sex Differ. 2024 Apr 23;15:37. doi: 10.1186/s13293-024-00611-5 (PMC11036720; doi:10.1186/s13293-024-00611-5)
Supplement: Supplementary file 13 — Supplementary Material 13 [file 13293_2024_611_MOESM13_ESM.docx]

Mar 28, 2024

Dr. Feng WANG

Shenzhen Key Lab of Neuropsychiatric Modulation, Shenzhen-Hong Kong Institute of Brain Science, Guangdong Provincial Key Laboratory of Brain Connectome and Behavior, CAS Key Laboratory of Brain Connectome and Manipulation, Shenzhen Institute of Advanced Technology (SIAT), Chinese Academy of Sciences (CAS)

Email: [feng.wang@siat.ac.cn](mailto:feng.wang@siat.ac.cn),

Web: http://bcbdi.siat.ac.cn/

Dr. Jill Becker

Editor-in-Chief

***Biology of Sex Differences***

**Manuscript ID BOSD-D-23-00276R1**

The manuscript entitled ***“Whole-brain connections of glutamatergic neurons in the mouse lateral habenula in both sexes”***.

Dear Dr. Becher,

We are grateful for receiving your decision letter dated Mar 14^th^, 2023, regarding our manuscript (BOSD-D-23-00276R1) entitled “*Whole-brain connections of glutamatergic neurons in the mouse lateral habenula in both sexes*” by Huang et al. We appreciate the editors and reviewers for their constructive comments and suggestions, which significantly improved the revised submission.

In response to the constructive feedback from the reviewer and editors, we have made several key revisions:

1. We have rectified grammar issues to ensure accuracy.

2. We have enhanced the clarity of the Methods and Results sections.

We have addressed the comments from the editors and reviewer#2 and made necessary revisions to our manuscript. Our point-by-point responses are provided below, with their comments *italicized* and our responses highlighted in blue. Textual changes in the revised manuscript are highlighted in red. We believe that the updated version is now suitable for publication in ***Biology of Sex Differences***.

***Editors' comments:***

Experts in the field have carefully reviewed your manuscript entitled, Whole-brain connections of glutamatergic neurons in the mouse lateral habenula in both sexes' (BOSD-D-23-00276R1). The reviewers have some minor concerns that require your attention before the paper can be accepted in its final form. The primary concern of reviewer 2 relates to grammar in the article, which I also find needs some work. Your methods and be clear and for that they need to be grammatically correct. I recommend that you have an expert in English, who also has knowledge of the subject matter help you with this revision. Shorter sentences and use of commas, where needed, would help with clarity.

Response: Thank you for your suggestions. We have thoroughly reviewed the manuscript and addressed the concerns raised by editors and reviewer #2 regarding grammar and clarity. We have also enlisted the help of an English expert with subject matter knowledge to ensure the revisions are made professionally. Additionally, we have ensured that the revised manuscript aligns with the *Biology of Sex Difference* journal style, including the required sections, and there have been no changes in authorship.

***Reviewers' comments:***

Reviewer #2: The authors have been very responsive to the critiques. There is an unavoidable issue of variance in the data, but the work is presented in ways that make this completely transparent. Although it is appreciated that 2 subjects have been added to each group, it is clear that these two have injection site locations that are less well targeted than the other 4 for both groups. So this is likely to have brought more variance, and may have eroded the possibility of identifying sex differences rather than strengthening the conclusions. However, all if this information is available for all readers to appreciate and consider for themselves. The data are also now interpreted and discussed more appropriately and with better appreciation of their limitations.

There are significant remaining concerns with grammar and clarity, especially in (but in no way limited to) the results section. For example, page 14, paragraph beginning on line 2 needs to be largely re-written for both grammar and clarity.

Response: Thank you for your suggestion. We have considered your suggestions and have made significant improvements to the clarity and grammar in the methods and results sections of our manuscript. Specifically, we have addressed the issues with the paragraph beginning on Line 3, Page 14.

Other suggestions:

Page 8, euthanized is generally used instead of sacrificed.

Response: Thank you for your suggestion. We have updated the word to “euthanized” in Line 10, Page 7 and Line 1, Page 8.

Page 9. The description should be how cells were selected for counting, not sections.

Response: Thank you for bringing this to our attention. We have updated the description to specifically address how cells were selected for counting. You can find the revised information in the Methods section, from Line 15 on Page 9 to Line 9 on Page 10.

Thank you for reviewing our manuscript for potential publication. We look forward to hearing from you at your earliest convenience.

Yours sincerely,

Feng WANG, MD, PhD.,

The Brain Cognition and Brain Disease Institute,

Shenzhen Institute of Advanced Technology,

Chinese Academy of Sciences;

Shenzhen Key Laboratory of Neuropsychiatric Modulation, Shenzhen-Hong Kong Institute of Brain Science,

Shenzhen 518055, China

Email: [feng.wang@siat.ac.cn](mailto:feng.wang@siat.ac.cn).
